# 40. NEW INSIGHTS ON THE ROLE OF NEUROINFLAMMATION IN SCHIZOPHRENIA PATHOPHYSIOLOGY FROM POST MORTEM AND ANIMAL STUDIES

**DOI:** 10.1093/schbul/sby014.163

**Published:** 2018-04-01

**Authors:** Tertia Purves-Tyson

**Affiliations:** Neuroscience Research Australia

## Abstract

**Overall Abstract:** Recent years have witnessed an explosion of clinical and preclinical effort aimed at understanding the involvement of neuroinflammation in schizophrenia (SCZ). The aim of this symposium is to present new and complementary data from human post mortem brain tissue and the rodent maternal immune activation (MIA) model, which together support the involvement of neuroinflammation in SCZ.

Tertia Purves-Tyson will present the first post mortem evidence that neuroinflammation in SCZ extends to the midbrain, a region critical for psychosis and cognitive deficits. She will show that: 1. gene expression of multiple pro-inflammatory cytokines is increased in the post mortem substantia nigra in SCZ compared to controls, in whose brains no such changes are seen; 2. as in the cortex, gene expression changes were found only in a subset of cases (~50%) of the SCZ cohort. She will also explore whether previously identified decreases in dopamine-related transcripts (transporters and receptors) in the substantia nigra in SCZ brains are related to the inflammation status. Ulrike Weber will show in the MIA model that the inflammatory changes identified in the midbrain of patients with SCZ may have a prenatal origin stemming from exposure to inflammation-related environmental insults. Remarkably mimicking the post-mortem data, MIA in mice increased brain pro-inflammatory cytokine gene expression, in not only the prefrontal cortex, but also in the ventral midbrain, and, similarly to humans, only in ~50% of offspring. She will also explore the relationship between changes in immune-related and dopamine-related gene expression in this brain region of MIA offspring. Anthony Vernon will show that MIA exposure on GD15 in rats leads to increased microglia density and soma size in the adult rat striatum and cingulate cortex. Strikingly, chronic haloperidol treatment at clinically comparable doses in adulthood interacts with prenatal MIA exposure, leading to further increases in both microglia density and soma size in both the striatum and frontal cortex. These data suggest adult antipsychotic exposure may increase neuroinflammation in the MIA model. Ina Weiner will report on the effects of subchronic low-dose risperidone treatment (RIS) in adolescence on neuroinflammation in MIA offspring, quantified using radiolabeled [3H]PK11195, a selective TSPO ligand and clinically comparable index of putative microgliosis. Compared to controls, [3H]PK11195 binding was increased in the hippocampus and frontal cortex of adult males and the hippocampus of females, with no changes in adolescence, partially mirroring the results of [11C]PK11195 in-vivo PET in SCZ patients. These increases were prevented in MIA offspring after RIS administration in adolescence, in parallel with prevention of brain volumetric reductions and cognitive deficits. Early intervention with RIS may decrease neuroinflammation and potentially underlie the preventive effects of RIS in the MIA model.

Taken together, these data support the involvement of neuroinflammation in SCZ and MIA model rodents. In particular, they suggest that high inflammatory profile, while distinguishing SCZ/MIA brains from control brains, may characterize only subsets of patients/MIA offspring, or may exist in all individuals but on an on-off basis, implying that preventive and current treatments may interact with neuroinflammation. Indeed, while APDs given to adult symptomatic offspring, interact with neuroinflammation to increase it further, early treatment with APDs in nonsymptomatic offspring prevents neuroinflammation. Evaluation of fluctuations in neuroinflammation over the lifespan and their interactions with treatment effects is a next step.

